# Halotolerant rhizobacteria *Pseudomonas pseudoalcaligenes* and *Bacillus subtilis* mediate systemic tolerance in hydroponically grown soybean (*Glycine max* L.) against salinity stress

**DOI:** 10.1371/journal.pone.0231348

**Published:** 2020-04-16

**Authors:** Humaira Yasmin, Sana Naeem, Murk Bakhtawar, Zahra Jabeen, Asia Nosheen, Rabia Naz, Rumana Keyani, Saqib Mumtaz, Muhammad Nadeem Hassan

**Affiliations:** Department of Biosciences, COMSATS University Islamabad (CUI), Islamabad Campus, Islamabad, Pakistan; Bhabha Atomic Research Centre, INDIA

## Abstract

Salt stress is one of the devastating factors that hampers growth and productivity of soybean. Use of *Pseudomonas pseudoalcaligenes* to improve salt tolerance in soybean has not been thoroughly explored yet. Therefore, we observed the response of hydroponically grown soybean plants, inoculated with halotolerant *P*. *pseudoalcaligenes* (SRM-16) and *Bacillus subtilis* (SRM-3) under salt stress. *In vitro* testing of 44 bacterial isolates revealed that four isolates showed high salt tolerance. Among them, *B*. *subtilis* and *P*. *pseudoalcaligenes* showed ACC deaminase activity, siderophore and indole acetic acid (IAA) production and were selected for the current study. We determined that 10^6^ cells/mL of *B*. *subtilis* and *P*. *pseudoalcaligenes* was sufficient to induce tolerance in soybean against salinity stress (100 mM NaCl) in hydroponics by enhancing plant biomass, relative water content and osmolytes. Upon exposure of salinity stress, *P*. *pseudoalcaligenes* inoculated soybean plants showed tolerance by the increased activities of defense related system such as ion transport, antioxidant enzymes, proline and MDA content in shoots and roots. The Na^+^ concentration in the soybean plants was increased in the salt stress; while, bacterial priming significantly reduced the Na^+^ concentration in the salt stressed soybean plants. However, the antagonistic results were observed for K^+^ concentration. Additionally, soybean primed with *P*. *pseudoalcaligenes* and exposed to 100 mM NaCl showed a new protein band of 28 kDa suggesting that *P*. *pseudoalcaligenes* effectively reduced salt stress. Our results showed that salinity tolerance was more pronounced in *P*. *pseudoalcaligenes* as compared to *B*. *subtilis*. However, a detailed study at molecular level to interpret the mechanism by which *P*. *pseudoalcaligenes* alleviates salt stress in soybean plants need to be explored.

## Introduction

Plant-growth-promoting bacteria (PGPB) are capable of enhancing plant growth by colonizing plant roots that can benefit the plant [1]. Among those, the halotolerant PGPB can withstand higher concentration of salt [2]. Halotolerant PGPB can promote plant growth and indirectly develop tolerance against stress by altering the selectivity of Na^+^, K^+^, and Ca^2+^ to sustain a higher K^+^/Na^+^ ratio, regulating the various antioxidant enzymes levels in the cells. These enzymes not only detoxify the harmful substances but also overcome the unwanted physiological changes under stress [3]. The halotolerant PGPB also adopt certain strategies like the induction of heat shock proteins, osmoprotectors and accumulation of proline that protect plants from stress conditions.

Bacterial 1-aminocycloprpoane-1-carboxylate (ACC) deaminase activity not only assimilates nitrogenous nutrients by breaking down the ethylene precursor ACC but also prevent the host plant from adverse effects of stress ethylene under biotic and abiotic stress conditions [4]. Moreover, Yasmin et al. [1] reported that IAA producing PGPR enhanced the nutrients uptake through modulating the root architecture in the drought stressed soil. Thus, PGPB can serve as a phytostimulator but potential traits of the PGPB can be altered genetically by different environmental stresses [5]. Consortium of newly isolated and already characterized PGPR will ensure their sustainable use in crop production [6].

Salinity is one of the foremost abiotic stresses that have severe impact on crop cultivation through limiting the environmental resources especially the physicochemical characteristics of soil [7]. Major contributors for increased salinity stress include firstly, poor cultural practices such as inappropriate fertilizer application and water management; secondly, climate change such as high evaporation with low rainfall [2]. Generally, salinity has negative effects on almost all stages of plant life cycle [8]. Thus, further research is being conducted to find new techniques to alleviate deleterious effects of salt stress and proper understanding of plant responses.

Ability of halophytes to cope up with the salt stress is related to various developed mechanisms to reduce the negative effects of salt stress. They include ion pumps, abscisic acid (ABA), osmoprotectants and ROS scavenging mechanism [2]. In order to develop strategies to increases salt tolerance in plants, a thorough understanding of salt tolerant mechanism against salinity stress is essential.

Soybean is cultivated worldwide due to its nutritional value and oil yielding characteristics [9]. It has a wide range of adaptations but as salt sensitive crop show limited seed germination, post germination growth, photosynthesis and yield reduction upto 40% due to salinity stress [10]. High salinity usually disturbs the nutrient balance in the soybean plants results in the ion toxicity and leads to osmotic stress [11]. The deleterious effects of salinity in soybean have been reduced by several conventional and non-conventional strategies such as seed priming, growth regulators, signalling molecules and salt tolerant varieties [12]. It has been proved from numerous studies that many bacterial strains belonging to different genera could efficiently reduce the deleterious effects of salinity stress [2].

In field settings, various environmental conditions greatly affect the use of potential rhizobacteria. To enhance agricultural growth, there is need of the isolation and identification of compatible PGPR strains to withstand the constantly varying environmental conditions. Therefore, we attempted to observe the role of PGPR in regulation of salinity tolerance in soybean. Several bacteria were isolated from saline soil and tested for their salt tolerance potential and among them *P*. *pseudoalcaligenes* and *B*. *subtilis* were selected. To the best of our knowledge, this is first report to show that *P*. *pseudoalcaligenes* can increase salinity tolerance in soybean plants by enhancing its defense responses; however, information is scanty about the role of *P*. *pseudoalcaligenes* in salinity tolerance of soybean. Therefore, we used the above mentioned bacterial strains for the assessment of physiological and biochemical responses of soybean to observe the protective mechanism under salt stress.

## Materials and methods

### Collection sites

In this study, saline soils cultivated with rice (*Oryza sativa*) and sugarcane (*Saccharum officinarum*) crops in Fatima Sugar Mills Limited, Fatima Sugar Research and Development Centre, Sanawan Kot Adu, Muzaffar Garh, Pakistan, were surveyed for the isolation of halotolerant rhizobacteria. Those rice and sugarcane plants were collected which showed better tolerance to salinity stress in saline fields.

### Soil sampling and physico-chemical properties

Root adhering soil samples were taken from rice (*Oryza sativa*) and sugarcane (*Saccharum officinarum*) crops grown under saline conditions. The collected roots with attached soils were kept in plastic bags for further processing. Physicochemical analysis of soil samples was done by Fatima sugar research and development centre, soil and water testing laboratories, Sanawan, Kot Adu, Muzaffar Garh, Pakistan. The soil samples were analyzed for their pH, EC, nitrogen, phosphorous, potassium, organic matter, sodium and sodium adsorption ratio (SAR) contents.

The soil pH ranged from 8.67–8.53 and highest organic matter (0.6%) was noted in rice fields. Soil samples showed variation in salinity levels, EC values was 4.35 dS/m for sugarcane field and 3.98 dS/m for rice field which is considered as high salinity levels for both crops and are a source of adverse effects on crop growth (Table 1).

**Table 1:** Characteristics of soil sampled for isolation of bacteria.

Field/Plot	pH	EC(dS/m)	Nitrogen (%)	Phosphorous (ppm)	Potassium (ppm)	Organic matter (%)	Sodium (ppm)	SAR
**Sugar cane Field**	8.67	4.35	0.023	6.78	140	0.51	1850	20.09
**Rice Field**	8.53	3.98	0.039	7.18	169	0.6	1560	18.38

### Isolation of rhizobacteria and halo-tolerance assay

Isolation of rhizobacteria was carried out on Luria Bertani (LB) media using serial dilution technique. Morphological features of the distinct colonies of each isolate were observed for different shapes, sizes, margins, surface, opacity and elevation. To evaluate the salt tolerance potential, all bacterial isolates were grown on LB media supplemented with various concentrations of NaCl (5, 10, 15 and 20%). The plates were inoculated with fixed volume of inoculum (OD = 0.05) incubated at 30°C for 7 days. The LB plates with 1% NaCl (w/v) were used as control [13].

### Plant growth promoting (PGP) characters of halotolerant bacteria

Screened salt tolerant bacterial strains were tested for PGP traits i.e phosphorous, potassium solubilization, siderophore, IAA production and ACC deaminase activity. All assays were performed in triplicates.

### Inorganic phosphate (Pi) and potassium (K) solubilization assay

Bacterial isolates were observed for phosphate solubilization by spot inoculation on Pikovaskaya’s media modified with tricalcium phosphate as substrate [14]. After incubation at 30°C for 7 days, plates were observed for halo-zones, and then solubilization index was recorded. Rhizobacterial isolates were tested for their potassium (K) solubilisation by spot inoculation on modified Aleksandrov medium containing mica powder as an insoluble source of K [15]. After incubation at 30°C for 3 days, formation of clear halo-zones was considered as K solubilisation positive.

### Siderophore production assay

Siderophore production was investigated by spot inoculation on chrome azurol S (CAS) blue agar plates followed by the method of Schwyn and Neilands [16]). After incubation for 72 h at 30°C, production of siderophores was indicated on the basis of discolouration (blue to orange-yellow) zones appeared around the colonies.

### Indole acetic acid (IAA) production assay

The IAA production by screened halotolerant bacterial isolates was observed following the method of Gordon and Weber [17]. The bacterial cultures were grown in LB broth for 24 h at 30°C. The 50 μL of overnight grown cells suspension was added in 5 mL of IAA production medium. These cultures were kept on shaking incubator (Irmeco GmbH, Germany) for 72 h at 32°C and 150 rpm along with control. A 1.5 mL bacterial culture was centrifuged at 12,000 rpm for 10 min to collect the cell free supernatant. The supernatant was mixed with equal volume of salkowski reagent and incubated for 1 h at 37°C. The appearance of pink to red colour indicated the production of IAA.

### ACC deaminase activity

The ACC deaminase activity of screened bacterial isolates was observed following the method of Jacobson et al. [18]. Single colonies of overnight grown bacterial strains were inoculated to 5 mL of sterile Dworkin and Foster (DF) salts minimal medium supplemented with ACC (3.0 mM) as sole source of nitrogen. The inoculated broth was incubated at 30°C for 72 h. The growth was checked daily recording to their optical density (OD) at 600 nm using spectrophotometer.

### 16S rRNA gene sequencing and phylogenetic analysis

Bacteria were identified at molecular level by sequencing 16S rRNA gene. The genomic DNA of bacterial isolates was extracted by CTAB method of Sambrook and Russell [19]. The 16S rRNA gene was amplified from the bacterial DNA using universal primers P1 (5’–CGGGATCCAGAGTTTGATCCTGGTCAGAACGAACGCT–3’) and P6 (5’–CGGGATCCTACGGCTACCTTGTTACGACTTCACCCC–3’). The PCR mixture was amplified in Thermocycler (Applied Biosystem). The PCR products were separated by electrophoresis in 1% agarose gels pre-stained with ethidium bromide. The amplified 16S rRNA gene was purified using Gene JET PCR Purification Kit (Thermo USA) and were sequenced using commercial services of Macrogen Seoul, Korea (http://macrogen.com/eng/). The sequences were annotated using the BioEdit software package [20]. The strains were identified using nearly complete sequence of 16S rRNA gene on Ez-Taxon (http://eztaxon-e.ezbiocloud.net). The phylogenetic tree was generated using the Phylogeny.fr platform and analysed using maximum-likelihood method implemented in the PhyML programme [21].

### Analysis of potential of halophiles for growth promotion and salinity tolerance in soybean

Seeds of Soybean genotype NARC 2011 were obtained from National Agricultural Research Centre (NARC), Islamabad. The seeds were surface sterilized by washing with 70% ethanol for one min followed by washing with sterile distilled water for three times. Thereafter, seeds were continuously stirred in 4% sodium hypochlorite (NaOCl) solution for 5 min and rinsed with distilled water for five times [22].

To conduct the bacterial treatment, screened salt tolerant bacterial strains SRM-16 (*P*. *pseudoalcaligenes* (MG733991)) and SRM-3 (*B*. *subtilis* (MG733990)) were used. The bacterial cell was used at the rate of 10^6^ CFU/mL. Overnight grown bacterial cultures were centrifuged at 15000 rpm for 3 min. Supernatant was discarded and equal volume of autoclaved distilled water was used to wash the pellet for three times. At the end, bacterial cultures were quantified on spectrophotometer at 600 nm in order to have the equal concentration of bacteria.

### Hydroponic experiment

Surface sterilized soybean seeds were placed on the autoclaved filter paper moistened with distilled water in petri plates [23]. After 3 days, the germinated seedlings were transferred to light for growth. After one week, the plants were subsequently transferred in continuously aerated nutrient solution in one-litre plastic pots.

The nutrient solution was Hoagland media based on the standard Hoagland recipe [24]. First, the plants were shifted to half strength Hoagland solution, then after three days, the Hoagland media were changed from half strength to full strength. Two-week-old seedlings were then inoculated with bacterial cells. After fourth day of inoculation, plants were subjected to six treatments (T1 = Control, 100 mM NaCl, *B*. *subtilis* (SRM-3), *P*. *pseudoalcaligenes* (SRM-16), *B*. *subtilis* (SRM-3) + 100 mM NaCl, *P*. *pseudoalcaligenes* (SRM-16) + 100 mM NaCl). The experiment was arranged as a completely randomized design with three replications. After two days of treatments, root and shoots were collected separately in replicates, instantly frozen in liquid nitrogen and kept at -80°C until processed for sodium dodecyl sulphate-polyacrylamide gel electrophoresis (SDS-PAGE).

After that, the full strength Hoagland media for rest of the plants were changed again. The inoculated plants continued to grow for seven days in the given bacterial and salt stress. At the end, all the six treatments were harvested for different phenotypic and biochemical parameters including antioxidant enzymes and non-enzymatic assays. All the samples were collected in the zipper bags and saved in -20°C freezer.

### Relative water content (RWC) of leaves

After 7 days of salt stress induction, the RWC of soybean leaves was accessed by the technique for Weatherley [25]. Following formula was used to calculate RWC.

RWC(%)=[(FW‐DW)/(FTW‐DW)]×100

While, RWC is relative water content; DW is dry weight of leaves; FW is fresh weight of leaves, and FTW is fully turgid weight.

### Analysis of photosynthetic pigments

Photosynthetic pigment of soybean leaves was extracted by the procedure of Burnison [26]. According to the protocol, small pieces of leaf sample (0.05 g) were incubated in 10 mL dimethyl sulphoxide (DMSO) for 72 h. Afterwards, the supernatant was collected and absorbance was measured on spectrophotometer (chlorophyll (chl) a (663 nm), chl b (645 nm) and carotenoids at 480 nm).

### Estimation of total protein

The total protein content of soybean leaves was quantified following the procedure of Lowry et al. [27]. Fresh leaves (0.1g) were crushed in phosphate buffer (pH = 7) and centrifuged for 10 min at 3000 rpm. The supernatant (0.1 mL) was collected and volume was raised with distilled water up to 1 mL followed by addition of same volume of the alkaline copper sulphate reagent. After shaking for 10 min, Folin’s reagent was added and mixture was incubated at 28±2°C for *30* min. The absorbance of each sample was noted at 650 nm. The concentration of total protein was quantified with reference to the standard curve of BSA (bovine serum albumin).

### Protein profiling of soybean leaves by SDS-PAGE (Sodium dodecyl sulphate- Polyacrylamide gel electrophoresis)

For one- dimensional separation of proteins, SDS-PAGE was performed according to the procedure of Laemmli [28]. Shoots samples were finely ground in liquid nitrogen and homogenized with 10 mL of 0.5 M Tris HCl, then filtered and incubated overnight at 4x. After that, samples were centrifuged at 14000 rpm (at 4°C) for 20 min, pellet was discarded and supernatant was saved. Supernatant (20 μL) was mixed with the loading dye (5 μL) and placed in the heat shock apparatus for 4 min at 95°C.

The electrophoresis plates of SDS-PAGE were filled with the 3/4^th^ of the resolving gel (15%) and allowed it to solidify for 15 min. After that 4% stacking gel (5 mL) was poured over the resolving gel. Samples (10 μL) and a protein marker (Fermentas, protein ladder) 5 μL were loaded in the wells. Then plates were placed into tank filled with electrode buffer, pH 8, and allowed to run at 100 V for 30 min and then on 150 for 1 h at 100 mA. After complete electrophoresis, gel was removed and first placed in the staining dye for 50 min and then shifted in the de-staining dye.

### Proline test

Proline content of soybean leaves was done according to the method proposed by Carillo and Gibon [29]. The fully expanded leaves (0.1 g) were crushed in ethanol (80%) and incubated in the water bath for 1 h at 80°C. Supernatant (0.5 mL) was collected after centrifugation at 12000 rpm for 10 min. After that, equal volume of distilled water and double the volume of 5% phenol were added in the supernatant. This mixture was kept for 1 h for incubation at the room temperature. Then, sulphuric acid (2.5 mL) was added and the absorbance of the solution was measured at 490 nm on spectrophotometer.

### Lipid peroxidation test

The quantity of lipid peroxidation of soybean leaves was determined by thiobarbituric acid assay following the protocol of Buege and Aust [30]. The 0.5 g of fresh leaves was grinded and homogenized with the 100 mM phosphate buffer and made the total volume 8 mL. Supernatant was collected after centrifugation for 20 min at 15,000 rpm at 4°C. In this enzyme extract (1.5 mL), 2.5 mL of reaction solution (5% Trichloroacetic acid (TCA) and Thiobarbituric acid (TBA) was added. Then, this solution was kept in water bath at 100°C for 30 min and then cooled at room temperature. After that, supernatant was collected by centrifugation at 4800 rpm for 10 min. The absorbance of supernatant was measured at 532 nm using spectrophotometer. For blank absorbance TBA and 5% TCA were used on spectrophotometer.

### Nutrient analysis by wet digestion

Nutrient analysis of leaves and roots was carried out according to the procedure of Thomas et al. [31]. All the samples were dried, ground into the fine powder and 10 mL of nitric acid-perchloric acid (2:1) was added to it and allowed to stand overnight. After preliminary digestion, the samples were heated at 150°C to 235°C, until the orange fumes converted to white fumes in the fume hood. When vapours got condensed by adding 2–3 mL deionized water, final volume was raised to 50 mL. The extract was filtered and analysed on atomic absorption spectrophotometer.

### Reactive oxygen species (ROS) scavenging enzyme activities analysis

#### Enzyme extract preparation

For sample preparation, 0.5 g each sample (shoots and roots) of the treatments was ground separately in 5 mL of 100 mM phosphate buffer) on the ice [32]. The homogenate were then centrifuged at 8000–13000 rpm for 20 min. The pellet formed was discarded and supernatant was saved at 4°C for performing biochemical assays.

#### Superoxide dismutase (SOD)

For SOD activity, the protocol of Beyer and Fridovich [33] was followed. The 0.025 mL of enzyme extract, 0.25 mL of H_2_O was mixed with 2.5 mL of reaction mixture. Two controls were also prepared as standard solutions containing 2.5 mL of reaction mixture and 0.25 mL H_2_O, one in 100% light and other in 100% dark. One set was placed in the light at 4000 lux for 20 min, while other control set was kept under complete dark conditions. The absorbance was read at 560 nm by spectrophotometer.

#### Catalase (CAT)

The activity of catalase enzyme in soybean leaves was performed by the protocol of Kumar et al. [34]. In the enzyme extract (0.1 mL), equal volume of 300 mM H_2_O_2_ and H_2_O, and 2.8 mL of 100% dilute 50 mM phosphate buffer was added. The absorbance of this reaction mixture was recorded at 240 nm at 0 min and 3 min.

#### Ascorbate peroxidase (APX)

The APX assay was done by the technique of Starlin and Gopalakrishnan [35]. In the enzyme extract (1 mL), 100% dilute 50 mM of phosphate buffer (2.7 mL), 7.5 mM ascorbate peroxide (0.1 mL) and 300 mM H_2_O_2_ (0.1mL) was added. Distilled water in place of enzyme solution in the reaction solution was used as blank. The absorbance was taken at 290 nm at the time interval of 0–60 s.

#### Peroxidase activity (POD)

The activity of POD enzyme was accessed by the protocol of Vetter et al. [36] altered by Gorin and Heidema [37]. The reaction solution comprised of 0.5 mL of enzyme extract, 1.5 mL of 0.05 M pyrogallol and 0.5 mL of 1% H_2_O_2_ was kept at 28 ± 2°C for 10 min in incubator. Variation in the absorbance was accessed at the intervals of 30 s, 1 min and 3 min at 240 nm using spectrophotometer. The peroxidase activity was quantified as variation in absorbance/min/mg of protein.

#### Polyphenol oxidase (PPO)

The PPO activity of soybean leaves was accessed following the procedure of Kahn [38]. Reaction mixture was prepared by adding the 200 μL enzyme extract, 1.5 mL of 0.1 M sodium phosphate buffer (pH = 6.5) and equal volume of 0.01 M catechol. The absorbance was taken on spectrophotometer at the time breaks of 30 s for 3 min at 496 nm. The PPO activity was introduced as variation in the OD 485 nm/min/mg of protein.

#### Phenylalanine ammonia lyase (PAL)

The activity of PAL enzyme of soybean leaves and roots was done following the procedure of Suzuki et al. [39]. Enzyme extract (30 μL), 3 mM L-phenylalanine which was made in 150 mM tris HCL buffer (pH = 8.5) (670 μL) and distilled water (300 μL) comprised the reaction solution. Optical density was recorded at 270 nm at the time intervals of 30 s for 3 min. The PAL activity was quantified as the average of the alteration in the absorbance by the time intervals.

### Data analysis

The data was analysed statistically by one-way analysis of variance (ANOVA) suitable to completely randomized design (CRD) and correlation coefficient using the software Statistix version 8.1. Mean values were compared by least significant difference (LSD) at P≤0.05 [40]. Heatmap for correlation coefficient was generated using web tool clustvis (https://biit.cs.ut.ee/clustvis/).

## Results

### Isolation and screening of salt tolerant bacterial strains

Forty four bacterial strains were isolated from salt affected soil samples of sugarcane and rice. All bacterial colonies exhibited different morphological attributes such as; surface, shape, edge/margin, elevation, opacity, and size etc. (S1 Table).

Out of 44 rhizobacterial isolates tested for halotolerance assay, all the isolates were able to tolerate 1 and 5% while SRM-1, SRM-3, SRM-4, SRM-9, SRM-14, SRM-16, and SRM-20 were able to tolerate salinity levels up to 10 and 15% NaCl concentration. Only 4 isolates (SRM-3_,_ SRM-9_,_ SRM-19 and SRM-20) tolerated up to 20% NaCl (S2 Table).

### Plant growth promoting (PGP) traits

On the basis of halo tolerance assay’s results, 4 isolates were tested for *in vitro* PGP traits i.e phosphorous and potassium solubilisation, siderophore production, IAA production and ACC deaminase activity. Phosphate and potassium solubilization was not detected by any of the bacterial isolate as shown in Table 2. Only two isolates were able to produce siderophore and formed clear zone around colonies in CAS-blue agar media. All the isolates were positive for ACC deaminase activity and IAA production in culture media.

**Table 2:** Higher salt concentration tolerance and PGPR activities of halotolerant bacterial strains.

**Bacterial Strains**	**Growth at 20% NaCl**	**Siderophore Production (SI)**	**ACC deaminase activity**	**IAA**
**SRM-3**	+	_	+	+
**SRM-9**	+	2.36	+	+
**SRM-16**	+	3.11	+	+
**SRM-20**	+	_	+	+

Plant growth promoting traits; + (plus) indicates presence;—(minus) indicates absence of respective traits tested

#### Identification of PGPR by 16s rRNA gene sequence analysis

**Fig 1:**
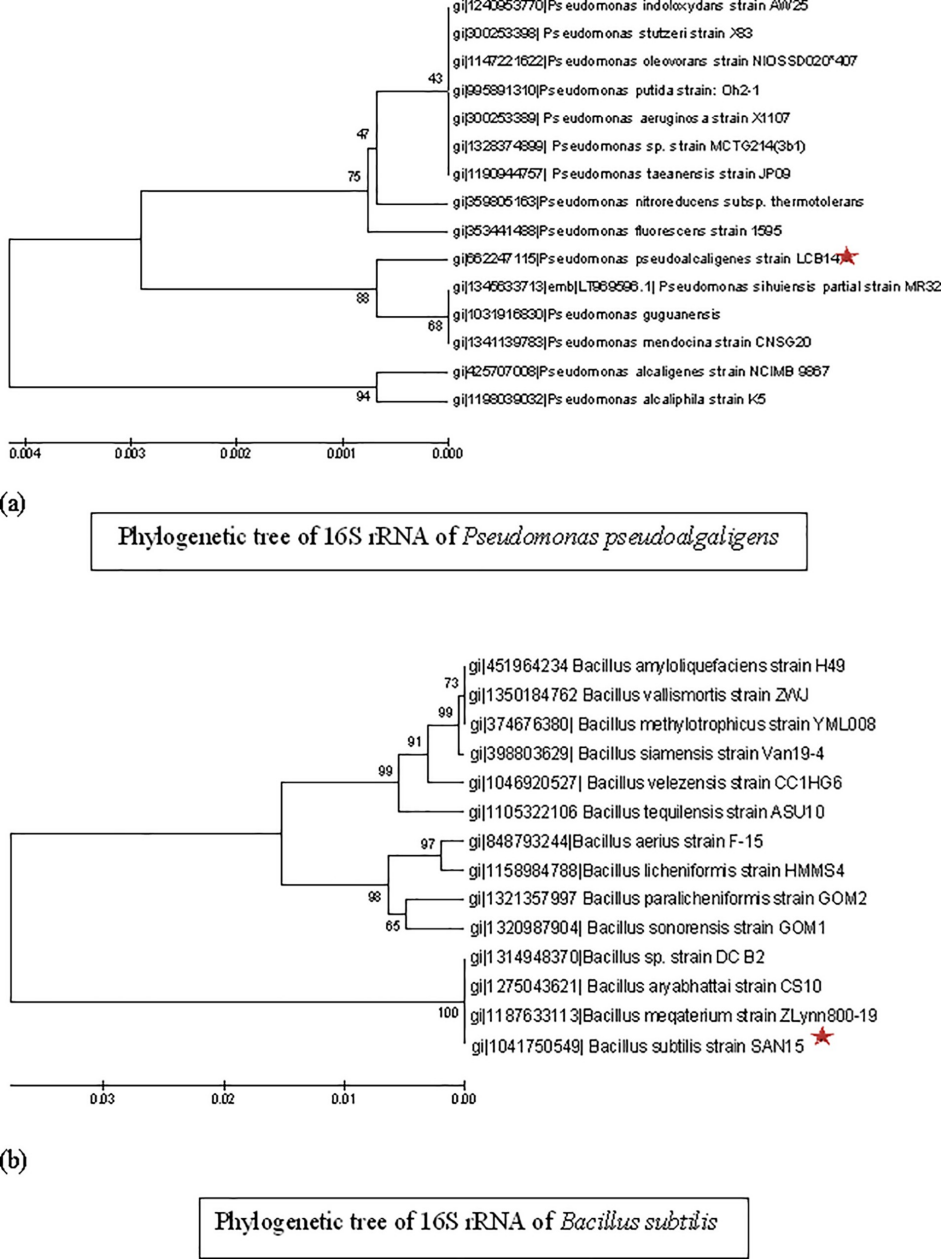
Phylogenetic analyses of a) *Pseudomonas pseudoalgaligens* SRM-16 and b) *Bacillus subtilus* SRM-3 on the basis of 16srRNA gene sequence analysis.

Identification of PGPR isolates was done by analysis of 16S rRNA gene sequence. On the basis of comparison of 1500bp sequences with the NCBI database the bacterial isolate SRM-3, SRM-9, SRM-16 and SRM-20 showed maximum similarity i.e 99% with *Bacillus subtilis*, 97% with *Bacillus megaterium*, 98% with *Pseudomonas pseudoalcaligenes*, 99% with *Bacillus* sp., respectively (Table 3). The Phylogenetic analysis based on 16S rRNA revealed that the *Bacillus subtilis* (SRM-3) falls in clade of species viz *Bacillus meqaterium and Bacillus aryabhattai and Bacillus sp*. *whereas P*. *pseudoalcaligenes* (SRM-16) falls in clade of species viz *P*. *sihuiensis*, *P*. *guguanesis*, *P*. *mendocina* (Fig 1).

**Table 3:** Identification of bacterial isolates on the basis of 16S rRNA gene sequence analysis.

Bacterial strains	Closest homologue (% similarity)	Accession number
**SRM-3**	*Bacillus subtilis* (99%)	MG733991
**SRM-9**	*Bacillus megaterium* (97%)	MG733989
**SRM-16**	*Pseudomonas pseudoalcaligenes* (98%)	MG736962
**SRM-20**	*Bacillus* sp. (99%)	MG733990

The % similarity is based on BLAST analysis

### Hydroponic experiment

#### Influence of salt tolerant rhizobacteria on the growth attributes of soybean (*Glycine max* L.) plants

Bacterial isolates *B*. *subtilus* (SRM-3) and *P*. *pseudoalcaligenes* (SRM-16) were screened and carefully chosen for the assessment of their influence on growth of soybean plants under hydroponic conditions when exposed to 100 mM NaCl stress.

**Fig 2:**
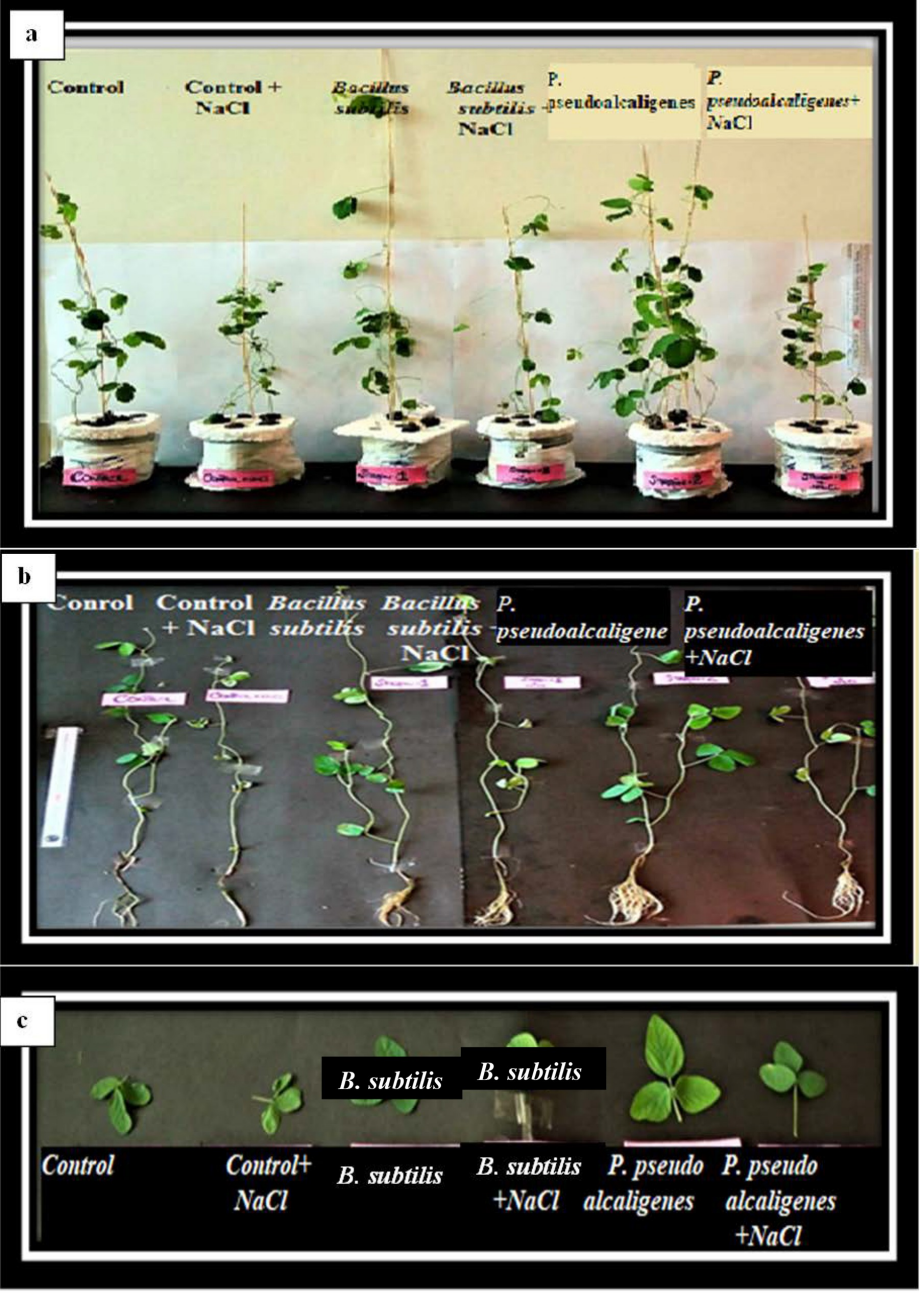
Salt treatments inhibited the a) shoot and b) root growth more intensively than that of PGPR treated plants. This was manifested in the salt-induced decrease in the weight and length of the roots and shoots (c) Salt treatment inhibited the leaf area of soybean plants more intensively than that of PGPR treated plants.

Exposure of salt stress to soybean was unfavourable for the growth of plants. The seedlings were lesser in mass, showed reduced vigour, premature senescence and wilted leaves. After 7 days exposure of soybean seedlings to salt stress, non-treated plants exhibited obvious effects of salt stress as compared to the PGPR treated seedlings as shown in Fig 2.

#### Influence of PGPR treatments on biomass of soybean grown under salt stress conditions

*Plant biomass*. All the inoculated treatments on soybean plants resulted in significant increase in shoot length, fresh and dry weight of shoots and roots and leaf area under salt stressed condition (Fig 2). Under salt stressed conditions, inoculation with bacterial isolate SRM-16 showed maximum increase in shoot length by 20%, fresh weight by 81% and dry weight by 48% when compared with non-inoculated control, respectively (Table 4).

**Table 4:** Effect of PGPR inoculation on shoot and root length and weight (fresh and dry) of soybean (*Glycine max* L.) under salt stress conditions.

Treatment	Shoot Length (cm)	Shoot Fresh Weight (g)	Shoot Dry Weight (g)	Root Length (cm)	Root Fresh Weight (g)	Root Dry Weight (g)
**Control**	60.90 ± 1.35 ^b^	2.08± 0.43 ^b^	0.3± 0.03 ^c^	17.9 ± 2.67^c^	0.11± 0.03 ^e^	0.11±0.03 ^a^
**Control + 100mM NaCl**	56.23 ± 1.61 ^c^	1.07 ± 0.37 ^d^	0.2 ± 0.02 ^d^	21.43± 2.07 ^b^	0.29 ± 0.02 ^c^	0.03 ± 0 ^c^
**SRM-3**	69.20 ± 1.31 ^a^	2.26 ± 0.50 ^b^	0.39 ± 0.02 ^b^	23.13±0.61 ^ab^	0.22±0.05 ^d^	0.04±0.02 ^c^
**SRM-3+ 100mM NaCl**	51.23 ± 5.49 ^d^	1.66 ± 0 ^c^	0.28 ± 0.02 ^c^	17.67 ± 1.30 ^c^	0.26±0.05 ^c^	0.04±0.02 ^c^
**SRM-16**	62.63 ± 3.33 ^b^	3.41 ± 0.51 ^a^	0.52 ± 0.03 ^a^	22.00 ± 3.46 ^b^	0.6±0.02 ^b^	0.06±0.04 ^b^
SRM-16. + 100mM NaCl	67.67 ± 2.65 ^a^	1.94 ± 0.11 ^bc^	0.29 ± 0.02 ^c^	24.97 ± 4.56 ^a^	0.65 ± 0.02 ^a^	0.05±0.02 ^bc^

Values are the means ± SE (n = 3). A different alphabet after each data within the same column represents the significant difference at 95% probability.

The data presented in Table 4 manifested that PGPR inoculated plants showed significant increase in root length under salt stress (Fig 2). However, SRM-16 represented 16% increase in root length as compared to the un-inoculated control in salt treated plants. Fresh and dry weight of root was significantly increased in bacterial inoculated plants as compared to control under salt stress. Treatment SRM-16 increased 124% fresh weight and 67% dry weight as compared to the control under salt stress (Table 3). The treatment SRM-16 increased leaf area by 174% as compared to the un-inoculated control under non stressed condition (Table 5).

**Table 5:** Leaf area and relative water content of the leaf under salt stress.

Treatments	Leaf Area (cm)	Relative Water Content (%)
**Control**	8.13 ± 1.94 ^c^	45.89 ± 0.98 ^b^
**Control + 100mM NaCl**	5.36 ± 1.59 ^d^	40.60 ± 2.18 ^c^
**SRM-3**	10.76 ± 0.54 ^b^	34.43 ± 2.10 ^d^
**SRM-3+ 100mM NaCl**	7.47 ± 0.03 ^cd^	51.20 ± 1.97 ^a^
**SRM-16**	8.90 ± 1.98 ^a^	49.71 ± 1.89 ^a^
**SRM-16. + 100mM NaCl**	14.69 ± 0.02 ^bc^	35.61 ± 0.64 ^d^

Values are the means ± SE (n = 6). A different alphabet after each data within the same column represents the significant difference at 95% probability.

#### Relative Water Content (RWC)

Plants treated with bacterial isolate SRM-3 under salt stress had significantly increased (26%) leaf water status as compared to the un-inoculated control under non stressed condition (Table 5).

#### Photosynthetic pigments

**Fig 3:**
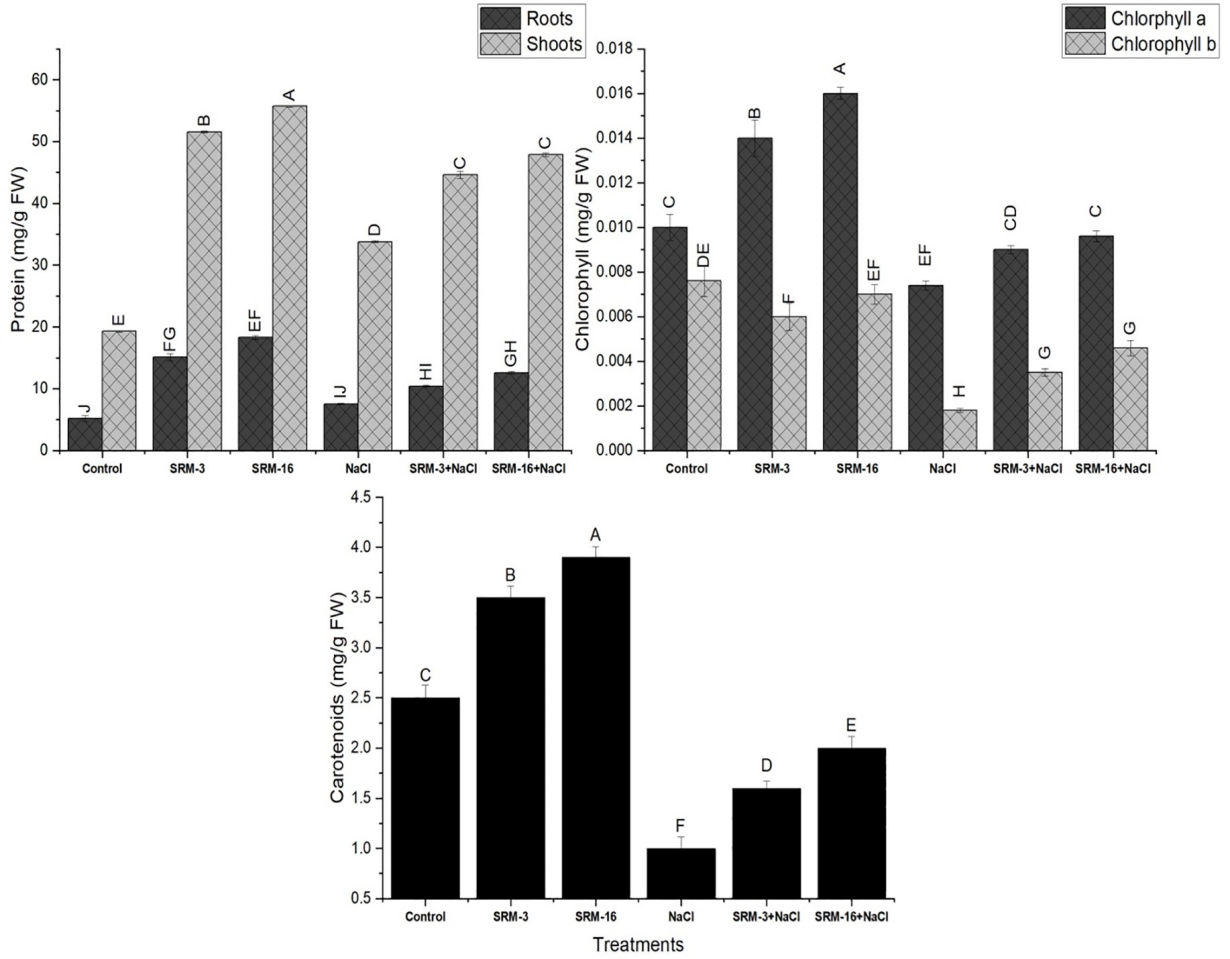
Effect of halotolerant *Bacillus subtilis* (SRM-3) and *Pseudomonas pseudoalcaligenes* (SRM-16) inoculation on the photosynthetic pigments and total protein contents of soybean (*Glycine max* L.) plants in control and salt stressed conditions. All the values are the mean of three replicates ± standard error of means. Different letters indicate statistically significant difference between treatments (*P*≤0.05) LSD is 2.176.

Chl a, Chl b and carotenoid content of soybean plant was significantly decreased by 26%, 76% and 60% respectively when a salt stress of 100 mM NaCl was induced in the hydroponic system as compared to the un-inoculated control plants. The halotolerant bacteria SRM-16 inoculation showed increase in Chl a and Chl b synthesis by 30% and 156% respectively as compared to the un-inoculated under salt stressed conditions (Fig 3). However, the maximum increase (100%) in carotenoids content was observed by SRM-3 inoculation as compared to the un-inoculated salt stressed plants (Fig 3).

#### Total protein content

All the PGPR inoculated treatments on soybean plants resulted in significant increase in proteins synthesis as compared to the un-inoculated plants. In the salt stressed plants, the protein synthesis was recorded 76% higher as compared to the control plants in shoots. Under the non-saline conditions, the maximum production of protein (190%) was observed by SRM-16 inoculation as compared to the non-saline control plants. However, under the salt stressed conditions, SRM-16 alleviated the effect of salt induced reduction of proteins in shoots and showed increase in the production of proteins by 42% as compared to un-inoculated salt stressed plants (Fig 3). In roots of soybean plant, under unstressed and salt stressed conditions, inoculation with SRM-16 showed maximum increased in protein synthesis by 251% and 67% as compared to un-inoculated control and salt stressed plants, respectively (Fig 3).

#### Lipid peroxidation

**Fig 4:**
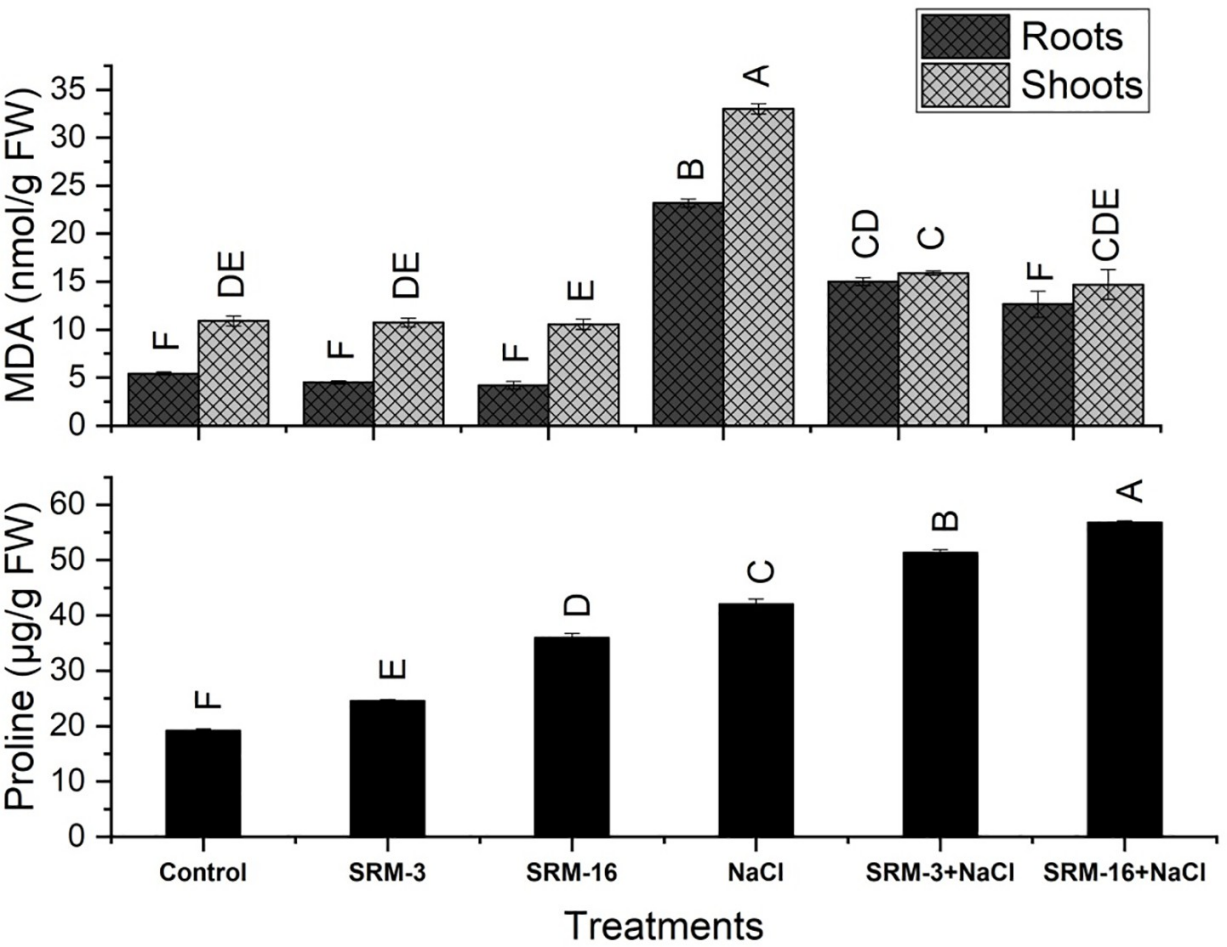
Effect of halotolerant *Bacillus subtilis* (SRM-3) and *Pseudomonas pseudoalcaligenes* (SRM-16) inoculations on MDA contents of roots and shoot, proline content of soybean (*Glycine max* L.) plants in control and salt stressed conditions. All the values are the mean of three replicates ± standard error of means. Different letters indicate statistically significant difference between treatments (*P*≤0.05) LSD is 2.176.

The PGPRs inoculation in the soybean plants showed significant reduction in MDA contents as compared to the salt stressed un-inoculated control plants. In salt stressed plants, the MDA level in plants was increased by 2-fold in shoots and 2.5 fold in roots as compared to the control plants. However, inoculation with the SRM-16 significantly decreased the level of MDA contents in shoots by 52% and in roots by 45% in salt stressed plants as compared to un-inoculated salt stressed plants (Fig 4).

#### Estimation of proline

The proline contents in shoots of soybean plants were significantly increased in the PGPR inoculated plants as compared to the un-inoculated control. The salt stressed plants showed 118% increase proline contents as compared to the control plants. Under unstressed conditions, maximal increase was manifested by SRM-16 treatment which represented 88% increase in proline synthesis as compared to the un-inoculated control plants. Under salt stressed treatments, the SRM-16 inoculated plants resulted in 35% increase in the proline synthesis as compared to the un-inoculated salt stressed plants (Fig 4).

#### Superoxide dismutase (SOD)

**Fig 5:**
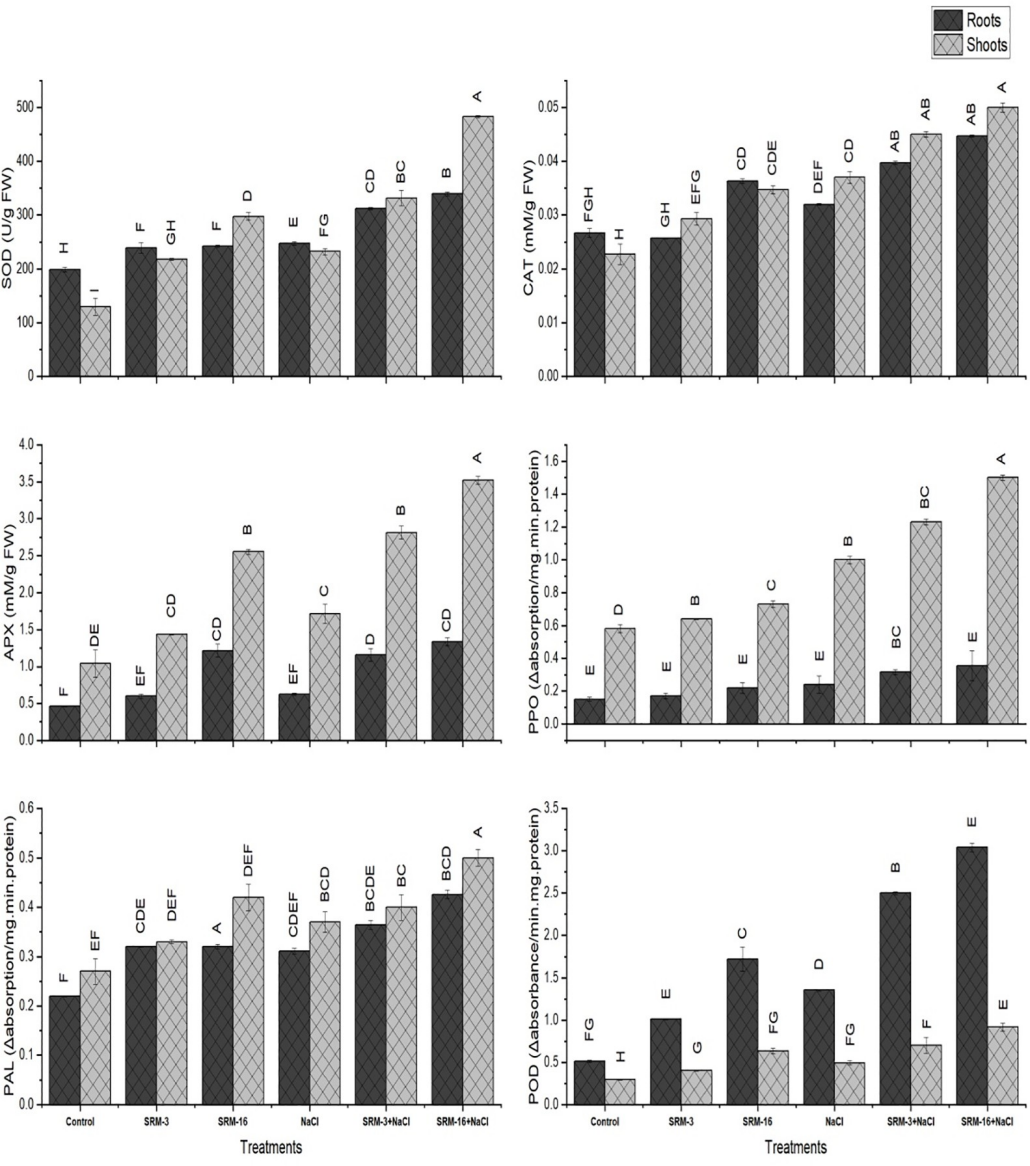
Effect of halotolerant *Bacillus subtilis* (SRM-3) and *Pseudomonas pseudoalcaligenes* (SRM-16) inoculations on enzymes activities of SOD, POD, CAT, APX, PPO, PAL of roots and shoots of soybean (*Glycine max* L.) plants in control and salt stressed conditions. All the values are the mean of three replicates ± standard error of means. Different letters indicate statistically significant difference between treatments (*P*≤0.05). LSD value is 2.176.

Under the unstressed conditions, SOD was significantly increased in PGPR inoculated plants as compared to the un-inoculated control in the roots and shoots. Under salt stress condition, treatment SRM-16 enhanced the SOD activity by 108% in the shoot as compared to the un-inoculated control. In roots, significant activity of enzyme was recorded by 12%, which was induced by SRM-16 inoculation as compared to the un-inoculated control (Fig 5).

#### Catalase (CAT)

In the non-saline PGPRs inoculated soybean plants, significant increase (20%) in the CAT activity was observed in shoots and 36% in roots with the SRM-16 inoculation as compared to the un-inoculated control plants. Bacterial isolate SRM-16 inoculation in the 100 mM NaCl stressed hydroponic system significantly increased the CAT activity by 13% and 40% in shoots and roots as compared to the un-inoculated saline plants, respectively (Fig 5).

### Ascorbate peroxidase (APX)

Bacterial isolate SRM-13 inoculation in the non-saline hydroponic system of soybean plants significantly increased the enzyme activity of APX by 65% in shoots and 87% in roots as compared to the un-inoculated control plants. Whereas, in the salt stressed hydroponic system, SRM-13 inoculation enhanced the enzyme activity by 1 fold and 1.5 fold in shoots and roots as compared to the un-inoculated salt stressed plants, respectively (Fig 5).

#### Peroxidase (POD)

Significant increase in the POD enzyme activity was observed by the inoculation of SRM-16 in the non-saline soybean plants by 1-fold in shoots and 2.5 fold in roots as compared to the un-inoculated control plants, respectively. Under the inoculated saline hydroponic system, maximum activity (83%) of POD enzyme was recorded by SRM-16 inoculation in shoots and 117% in roots as compared to the un-inoculated saline plants as shown in the Fig 5.

#### Phenylalanine ammonia lyase enzyme (PAL)

Under the PGPRs inoculated control soybean plants, significant activity in the PAL enzyme was observed by the SRM-16 inoculation. The SRM-16 inoculated non-saline plants increased the PAL activity by 7% in the shoots as compared to the un-inoculated control plants. However, 35% increase in the enzyme activity was observed by SRM-16 inoculation as compared to the un-inoculated saline plants (Fig 5). In the roots of soybean plant, under unstressed and salt stressed conditions, inoculation with SRM-16 showed maximum increased in PAL activity by 18% and 8% as compared to the un-inoculated control and salt stressed plants, respectively (Fig 5).

#### Polyphenol oxidase (PPO)

The PPO activity in the shoots of soybean plants was significantly enhanced by the treatments of PGPRs inoculation. Under the unstressed inoculated plants, SRM-16 increased the enzyme activity by 30% in shoots and 47% in roots as compared to the un-inoculated control plants. Under the salt stressed inoculated plants, the PPO activity was significantly enhanced by 50% in shoots and 48% in roots, which was induced by SRM-16 as compared to the un-inoculated salt stressed plants (Fig 5).

#### Nutrient analysis (Na^+^ and K^+^)

**Fig 6:**
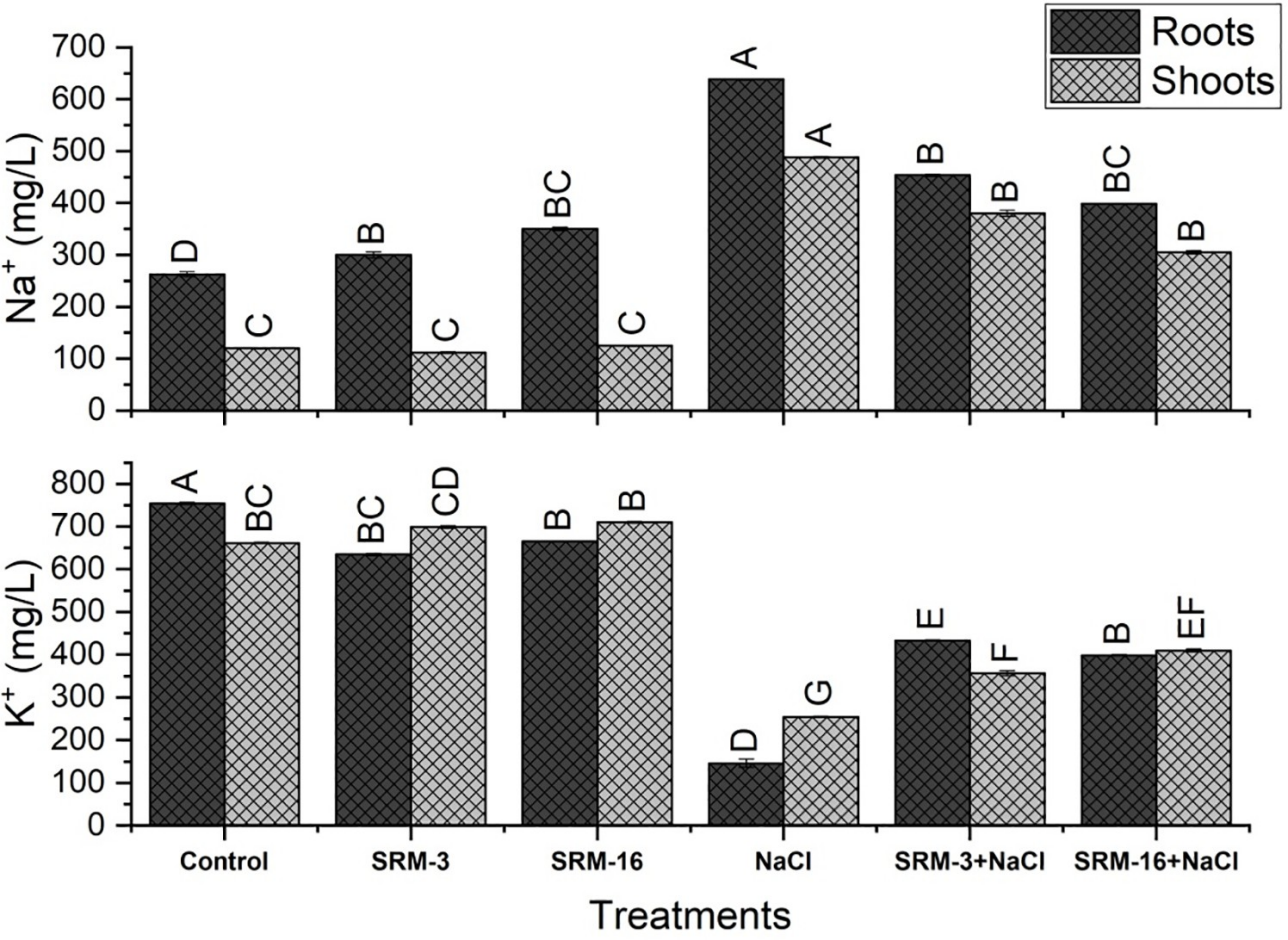
Effect of halotolerant *Bacillus subtilis* (SRM-3) and *Pseudomonas pseudoalcaligenes* (SRM-16) inoculations on Na^+^ and K^+^ contents of roots and shoots of soybean (*Glycine max* L.) plants in control and salt stressed conditions. All the values are the mean of three replicates ± standard error of means. Different letters indicate statistically significant difference between treatments (*P*≤0.05). LSD value is 2.176.

All the treatments significantly modulate the concentration of Na^+^ and K^+^ in the soybean plants grown hydroponically under the controlled and stressed conditions. Under the salt stressed conditions, Na^+^ concentration was significantly increased by 3-fold in shoots and 1.5 fold in roots as compared to the control plants. However, under the PGPRs inoculated salt stressed plants, significant decrease in the Na^+^ concentration was manifested by treatment with SRM-3 inoculation that represents 22% decrease in shoots and 23% decrease in the roots as compared to the un-inoculated salt stressed plants (Fig 6).

The K^+^ concentration under the salt stressed soybean plants was significantly reduced by 56% in both the shoots and roots as compared to the control plants. Under the PGPRs inoculated salt stressed conditions, maximum increase (61%) in the K^+^ concentration was observed by the SRM-16 treatment in shoots. In roots, SRM-3 inoculation showed 197% increase as compared to the un-inoculated salt stressed plants (Fig 6).

### Heatmap responses of Pearson’s Correlation Coefficient (r) for the antioxidant enzymes, stress determinants and Na^+^ ion concentration from soybean seedlings inoculated with PGPR under salt stressed condition

**Fig 7:**
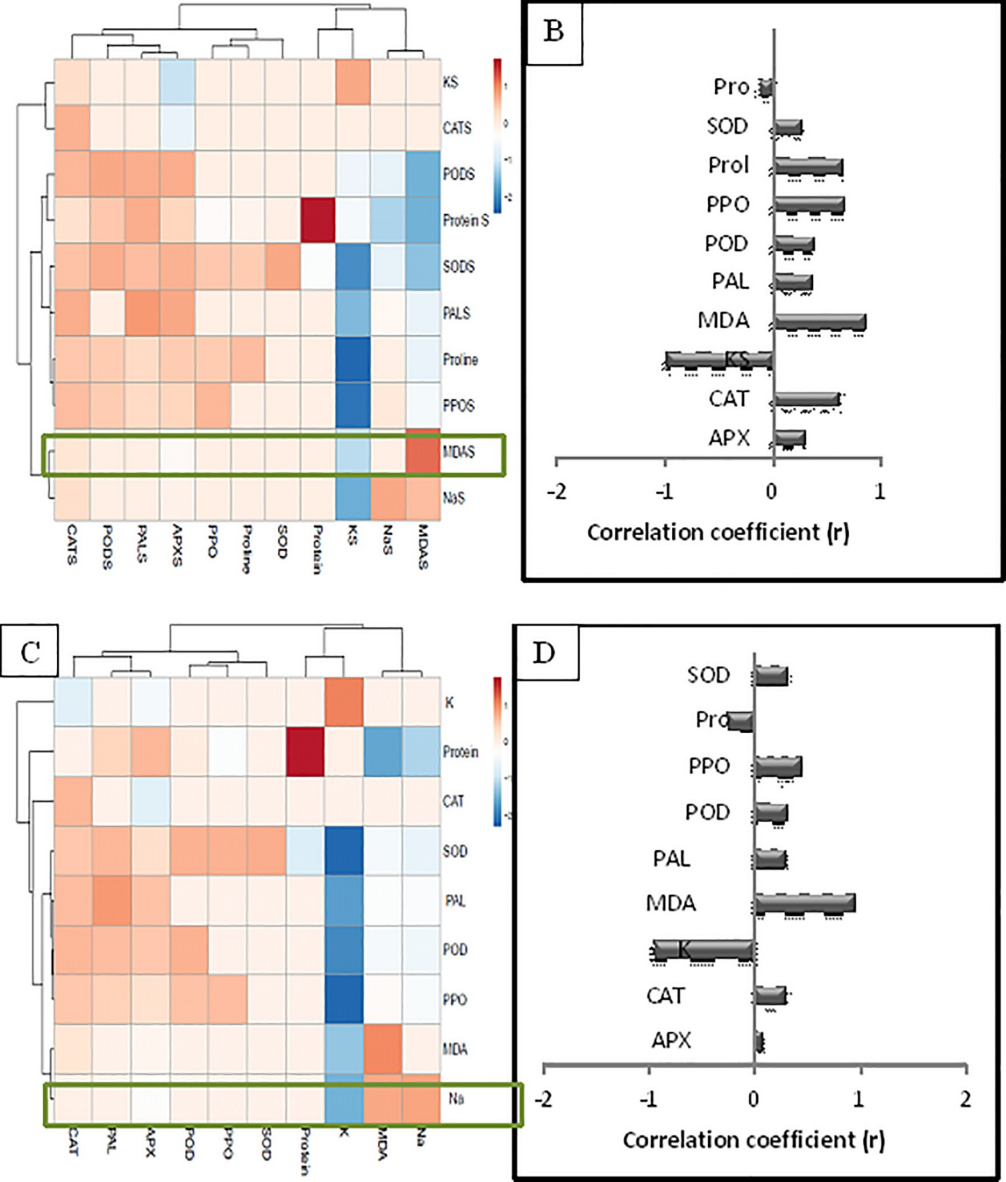
Heat map responses of Pearson’s correlation coefficient for the targeted antioxidant enzymes, stress determinants and ions (Na^+^ and K^+^) of shoots and roots of soybean treated with PGPR grown under salinity stress. Electrophoretic pattern of proteins in soybean leaves inoculated with PGPRs under salt stress by SDS-PAGE.

With respect to the salinity stress state of the soybean seedlings, the data for heat map were classified as root and shoot, and each group show positive correlations. A comparative analysis of the factors related to Na^+^ concentration (presented by green boxes) suggested that shoots and roots have a positive correlation with SOD, Proline, PPO, POD, PAL, MDA, CAT and APX activities and a negative correlation was observed with K^+^ ions and protein content (Fig 7B and 7D).

**Fig 8:**
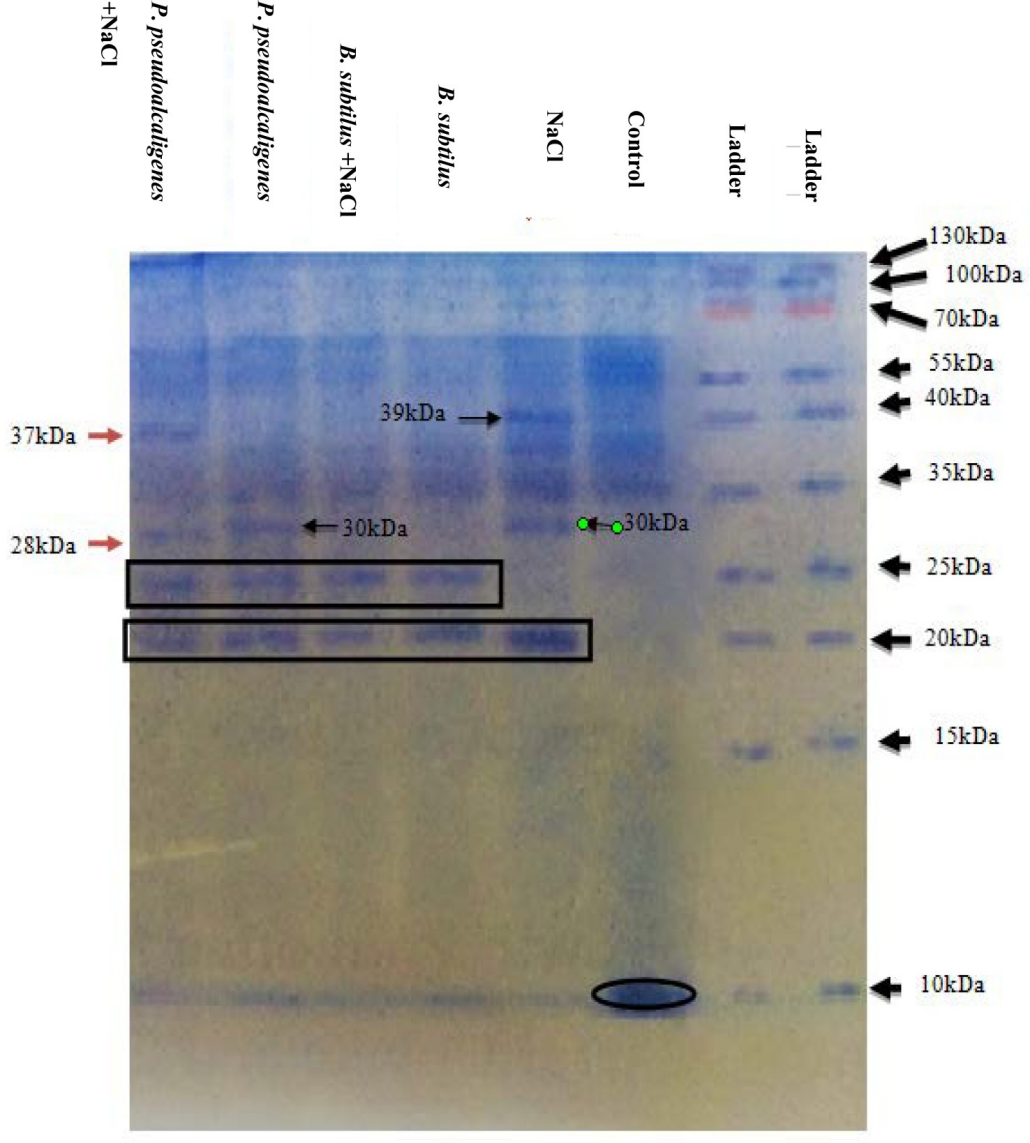
SDS-PAGE gel analysis of proteins extracted from soybean plants. Protein bands were visualized using Coomassie brilliant blue staining gels from samples treated with control, 100 mM NaCl, *B*. *subtilis*, *B*. *subtilis* + 100 mM NaCl, *P*. *pseudalcaligenes*, *P*. *pseudalcaligenes*. + 100 mM NaCl, respectively. Protein bands that showed significant changes under salt stress are labelled.

SDS-PAGE analysis clearly indicates that the NaCl and PGPRs treatments induced alterations in protein banding pattern in the leaves. The 100 mM NaCl treatment induced a new banding pattern of the 39kDa and 30kDa proteins in the gel. A band of approximately 28kDa was only appeared in SRM-16 inoculated saline plants. Another band of approximately 30kDa was observed in un-inoculated saline and SRM-16 inoculated non-saline plants. Protein band of 25kDa was synthesized in all the PGPRs treated plants saline and non-saline and absent in control and un-inoculated saline plants. Moreover, we observed that the 20 kDa protein appeared in NaCl treated control and all SRM-3 and SRM-16 inoculated plants except the un-inoculated control plants. The intensity of this new band increased in the salt stressed SRM-3 and salt stressed SRM-16 inoculated plants as indicated in Fig 8. However, the protein band with a molecular weight of 10kDa that was observed in control plants is disappeared at 100 mM NaCl and PGPRs treatments.

## Discussion

Salinity is one of the major factors limiting the productivity of agricultural crops with severe effects on germination, plant vigour and crop yield [41]. Soybean is categorized as a salt sensitive crop, so during the onset of salt stress within a plant, all the developmental processes are affected [42]. This results in reduced growth rate and nutritive quality of soybean seeds. The halotolerant PGPRs have been reported to promote plant growth as well as mitigate salinity stress [43, 44].

We performed *in vitro* screening of 44 bacteria isolated from salt affected rhizosphere soil of rice and sugarcane and found that *P*. *pseudoalcaligenes* (SRM-16) and *B*. *subtilis* (SRM-3) possess the highest salt tolerance properties. We also studied the role of halotolerant *P*. *pseudoalcaligenes* and *B*. *subtilis* in soybean growths with and without salt stress conditions. Praveen et al. [45] suggested that *B*. *subtilis* and *P*. *pseudoalcaligenes* are able to withstand the salt and drought stress and these isolates have good competitive abilities to perform well in the rhizosphere of the soil. However, information is scanty about the role of halotolerant *P*. *pseudoalcaligenes* in salinity tolerance of soybean. The screened halo-tolerant strains were able to produce IAA, siderophores and showed ACC deaminase activity. The ACC deaminase production has been considered as one of the major mechanisms used by PGPR to reduce the level of ethylene production in biotic and abiotic stresses in plants. The ACC is the precursor of hormone ethylene in plants. We found that these plant-associated microorganisms containing ACC deaminase played an important role in conferring tolerance to abiotic stresses by reducing the adverse effects of ethylene production [46].

Therefore, in our study, we determined the ability of *P*. *pseudoalcaligenes* and *B*. *subtilis* to show significant tolerance to salt stress in hydroponically grown soybean seedlings by measuring ROS-scavenging enzymes, photosynthetic pigments, MDA, proline, PPO, PAL, ion accumulation and protein profiling. Inoculation with these bacteria not only induced significant changes in the activity of different ROS-scavenging enzymes but also enhanced the growth and photosynthetic efficiency of inoculated soybean plants, which proved could be a reason for the tolerance of abiotic stress [43]. It has been observed that when bacteria make association with the plants, defence system of plant get activated and increased its antioxidant scavenging machinery. Inoculation-induced-alteration in antioxidant activity has also been reported by El-Esawi et al. [47] and Mesa-Marín et al. [48]. These strains can be efficiently deployed in extreme environments. Our results showed that growth promotion and salinity tolerance is more pronounced by inoculation with *P*. *pseudoalcaligenes* as compared to the *B*. *subtilis*. The results of this research showed that the microbial inoculations in plants under the salt stress have the cumulative effect on activities of enzymes [49]. Plant biomass and height both are important traits to increase growth and yield of plants. Variation in the patterns of growth enhancement has been observed when plants are treated with different or even same bacterial species. In this study *Bacillus* treated plants showed maximum height while *P*. *pseudoalcaligenes* inoculated plants showed higher biomass as compared to the control. For crop improvement, more emphasis was given on increasing biomass as compared to height to avoid lodging, high yield and complete maturation of fruit [50].

One of the prime responses of salt stress is that it slows down the efficiency of water uptake by the plants and disturbs the osmotic balance leading to apoptosis. In this situation, the compatible osmolyte proline activated and tried to mitigate the effects of salt [2]. Our results showed that the plants inoculated with the *P*. *pseudoalcaligenes* recorded significantly higher proline level and reduced malondialdehyde under salt stressed conditions in comparison to un-inoculated salt affected plants. Previous studies also reported the similar results in rice cultivar that the overexpression of the genes that are related to soluble sugars and proline, due to the upregulation of proline synthesis, was observed under the salt stress [51]. These results support the fact that proline content enhanced actively in defense response and also up regulated by PGPRs. Induction or activation of proline synthesis from glutamate or decrease in its utilization in protein synthesis or enhancement in protein turnover may result in the increase of proline synthesis. Thus, proline may be the major source of energy and nitrogen during stress metabolism and accumulated proline apparently supplies energy for growth and survival and tolerance during salt stress [52].

Melondialdehyde is a product of membrane oxidation and is a marker of the extent of membrane damage due to the salt stress [53]. In our results, less MDA level was recorded in control plants and increased amount was recorded in the salt affected plants, however, reduction was recorded in both the PGPRs inoculated salt affected plants in roots and shoots. These findings are correlated with the earlier observations of Bharti et al. [54] and AbdElgawad et al. [55] who concluded that reduced lipid peroxidation content is better for the salt resistance mechanism

Soybean seedlings showed adverse symptoms under salinity stress like yellowing, wilting and withering of leave. These morphological changes in the leaves are correlated with decreased photosynthesis. Photosynthesis is not only reduced by the stomatal closure, but also to non-stomata factors, these are the denaturation of photosynthetic enzymes, due to the ionic stress and reduction in the chlorophyll a, b and carotenoids contents [56]. In our study, significant reduction in photosynthetic pigments (chlorophyll a, chlorophyll b and carotenoids) was observed in salt affected plants than the control plants. This may due to the poor availability of CO_2_ or the destruction of photosynthetic apparatus by higher activity of chlorophyllase enzyme [57]. The phenotypic alterations in the leaves of soybean under salt stress were correlated with the reduced chlorophyll and carotenoid content (Fig 2). However, in this study, PGPR treatments showed the enhance synthesis of these pigments in salt stressed plants. Up till now, 6610 proteins have been identified in soybean plants that activates in the NaCl stress response. Among them, 278 proteins and 440 proteins up regulates in roots and shoots respectively [58]. The proteome level in the salt stressed plants is reduced than the control plants. PGPRs inoculated plants showed enhanced synthesis of defense protein in salt affected situations, indicating that *Bacillus subtilis* and *P*. *pseudoalcaligenes* have positive influence on the proteome regulatory system of plants [59, 60]. Hence, it may be correlated that PGPRs show interaction at either metabolic or molecular level. Results of our study are correlated with the findings of El-Esawi et al. [47] who reported a significant increase in salinity tolerance in soybean with the inoculation of halotolerant strain *Bacillus firmus* SW5.

The results of the current experiment showed significantly high Na^+^ concentration in the plants grown in salt stressed conditions both in shoots and roots but when these salt affected plants inoculated with the *B*. *subtilis* and *P*. *pseudoalcaligenes* strains, the Na^+^ concentration seemed to be decreased than the control saline plants. In the control plants, high K^+^ concentration was observed. These results are similar with the findings of AbdElgawad et al. [55] who found significant accumulation of Na^+^ concentration in both leaves and roots of maize plants grown under salinity stress. Less sodium amount was normally accumulated in soybean shoots and roots, basically due to higher rates of net ion transported from roots to shoots. In this study, under saline conditions, roots accumulated more Na^+^ than the shoots. This indicates that Na^+^ transportation from roots to shoots was accelerated when NaCl concentration was increased. A negative correlation between Na^+^ concentration and salt tolerance was also observed by [61] in soybean. In the PGPR inoculated plants, less leakage of K^+^ and less uptake of Na^+^ was observed than stressed plants. Our findings revealed that the PGPR regulated some of the key defense enzymes that induce the balanced cellular conditions by reducing intracellular Na^+^ concentration and maintained high K^+^ concentration in the cell [62].

ROS are reduced or the excited form of molecular oxygen present in the cell [63]. They have dual role in plants and are necessary for many signal transduction process in the cell but also toxic by-products of aerobic metabolism [64]. When the salt stress applied, it increased the ROS at toxic level, which are scavenged by antioxidant enzymes [65]. In our research, we studied the extent to which the antioxidant enzymes produced to scavenge the ROS. Our results are in accordance with El-Esawi et al. [47] who showed significant increase in antioxidant enzymes activity in salt stressed un-inoculated treatment as compared to the non-stressed control. The SOD acts as a first line of defense to encounter the abiotic stress and is very efficient in scavenging the superoxide radicles [66]. In our results, increased SOD activity was observed in salt stress treatments. However, its level was significantly increased in PGPR inoculated salt affected plants than salt affected plants to scavenge ROS to protect the plant from ionic and osmotic stress related damage [67]. The H_2_O_2_ accumulation by SOD acts as a secondary messenger for the upregulation of other defense enzymes such as CAT, POD, and APX [68]. The CAT, APX and POD activates as a second line of defense. It showed that acclimation of these enzymes correlates with the products of SOD [68]. Here, we found that higher activities of the antioxidant enzymes CAT, APX, POD results in the enhanced salt tolerance in soybean and *P*. *pseudoalcaligenes* have been found to mediate more salt resistance by regulating the ROS-scavenging enzymes [69].

Phenylalanine ammonia lyase (PAL) is a defense enzyme that activates upon the deficiency of important nutrient like nitrate. The PAL showed significant upregulation in *P*. *pseudoalcaligenes* inoculated control and salt stressed plants. Nitrate deficiency could occur under salt stress conditions due to their interference in uptake by Cl^**-**^ ion on certain systems of membrane transport in cowpea [70]. The Cl^-^ ion competes with nitrate for the binding site of the transporter. So to overcome the nitrate deficiency, PAL enzymes activate and convert the accumulated amino acid L-phenylalanine to ammonia [71]. The PAL also induces their upregulation in oxidative stress. Whenever, plant encountered the abiotic stress, phenolic compounds are produced to suppress the oxidative stress. Phenylpropanoid compounds are the precursors of wide range of phenolic compounds and PAL catalyses these reactions in plants [72].

The PPO is widely present in plant and oxidizes the phenols to quinones and also involved in the antioxidant defense response against biotic and abiotic stress [73]. In our results, less activity of PPO was recorded in the control plants while salt stressed plants showed the upregulation in its production as compared to the control plants. Whereas, in the PGPR inoculated salt stressed plants, enzyme activity was significantly enhanced than the un-inoculated plants. Phenolic compounds are the quencher of reactive oxygen species; therefore, PPO is indirectly involved in suppressing the oxidative stress [74]. In our results, the enzymatic activity in roots was not sufficiently increased in stress response as compared to shoots. It may be due to the fact that the substrate of the products of PPO is localized in the vacuoles and chloroplast, and PPO is residing in the thylakoid membrane, therefore, most of the activity of this enzyme was observed in the leaves and shoots areas of the plants [75].

Electrophoretic pattern of soybean plants grown under the saline conditions with the PGPRs inoculation, gives the information about the changes at proteome level. Control plants showed the normal banding pattern, however it was altered in the NaCl stressed and PGPRs inoculated plants.

A band of 10kDa was down regulated in salt and PGPRs treated soybean plants, this might be due inhibition or unavailability of sources in stress conditions to transcribe the protein. New bands of 20kDa and 25kDa appeared in all the salt and PGPRs treated plants, this indicated that these proteins might be translated to cope with stress conditions [76]. A protein band, with the molecular weight of 28kDa appeared only in *P*. *pseudoalcaligenes* inoculated salt stressed plant. This protein is specifically related to defense and regulatory mechanisms of soybean plants that is induced by *P*. *pseudoalcaligenes* only in response to salt stress. These results are in accordance with Vaishnav et al. [77] who found and characterize plant proteins of 28KDa induced by *Pseudomonas simiae* strain AU in soybean under salinity stress. This 28KD protein band was identified as vegetative storage protein (VSP) by MALDI-MS/MS. Furthermore, expression analysis by western blotting confirmed that the vegetative storage protein (VSP) were significantly up-regulated by the exposure to AU strain and played a major role in induced systemic tolerance. The VSPs are reported to regulate and respond by external stimuli, including salt stress, water deficiency, wounding, methyl JA (MeJA), sugars and light [78, 79]. The function of VSP is Na^+^ homeostasis, N accumulation and mobilization, acid phosphatase activity and to maintain plant growth under stress condition. In this study, *P*. *pseudoalcaligenes* develop the tolerance against the salinity by inducing VSPs. Our results, showing lower uptake of Na^+^ ions in bacterial-treated seedlings suggested that VSPs regulate sodium transporters and modulate Na^+^ ion homeostasis resulting in tolerance against salt stress. Gomathi and Vasanta, [80] also identified the 15, 28 and 72 kDa polypeptides present in the PGPR induced salt effected plants, they explained that these proteins are the salt shock proteins (SSP), which are absent in the salt sensitive species and found in the salt tolerant plants and these findings support the results of the current study.

## Conclusion

In conclusion, *P*. *pseudoalcaligenes* and *B*. *subtilis* promoted plant growth and induced tolerance in soybean plant from damage to salt stress. *P*. *pseudoalcaligenes* appeared to confer more resistance to salt by inducing higher level of key defense mechanisms in salt stressed conditions. It also activated the osmoregulators and ROS scavenging enzymes to reduce reactive oxygen species. These bacterial strains can help in the reclamation of salt effected arable land. However, further evidences are needed to verify the exact mechanism including the identification of stress responsive proteins, through which these particular PGPR mediate the tolerance against salt stress in soybean plants. Effect of combined application of both potent PGPR strains and their application at the field level needs to be further studied.

## Supporting information

S1 Dataset (XLSX)

S1 Raw image (JPG)

S1 Table Morphological characteristic of bacterial isolates from saline soil of rice and sugarcane. (DOCX)

S2 Table Relative growth of bacterial strain on varying NaCl concentrations. (DOCX)
