# Supplementary figures and images for: Halotolerant rhizobacteria Pseudomonas pseudoalcaligenes and Bacillus subtilis mediate systemic tolerance in hydroponically grown soybean (Glycine max L.) against salinity stress
Source: PLoS One. 2020 Apr 16;15(4):e0231348. doi: 10.1371/journal.pone.0231348 (PMC7162512; doi:10.1371/journal.pone.0231348)

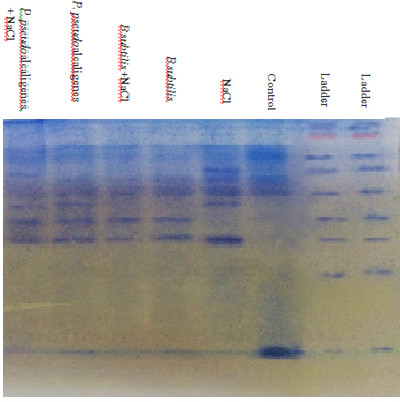

Supplement: S1 Raw image — (JPG) [file pone.0231348.s002.jpg]
