# Supplementary material for: Halotolerant rhizobacteria Pseudomonas pseudoalcaligenes and Bacillus subtilis mediate systemic tolerance in hydroponically grown soybean (Glycine max L.) against salinity stress
Source: PLoS One. 2020 Apr 16;15(4):e0231348. doi: 10.1371/journal.pone.0231348 (PMC7162512; doi:10.1371/journal.pone.0231348)
Supplement: S1 Table — (DOCX) [file pone.0231348.s003.docx]

**Supplementary Table**

**Table: S1. Morphological characteristic of bacterial isolates from saline soil of rice and sugarcane**

| **S. No.** | **Surface** | **Shape** | **Edge/Margin** | **Elevation** | **Opacity** | **Size (mm)** | **Host plant specie** |
| --- | --- | --- | --- | --- | --- | --- | --- |
| **SRM-1** | Glistening | Round | Entire | Raised | Opaque | > 5 | sugarcane |
| **SRM-2** | Glistening | Round | Entire | Raised | Opaque | > 5 | sugarcane |
| **SRM-3** | Glistening | Round | Entire | Raised | Opaque | 5 | sugarcane |
| **SRM-4** | Glistening | Round | Entire | Raised | Opaque | > 1 | sugarcane |
| **SRM-5** | Dull | Irregular | Lobate | Growth into Medium | Opaque | 3-5 | sugarcane |
| **SRM-6** | Smooth | Irregular | Lobate | Flat | Opaque | 5 | sugarcane |
| **SRM-7** | Smooth | Round | Entire | Raised | Opaque | > 5 | sugarcane |
| **SRM-8** | Wrinkled | Irregular | Lobate | Flat | Opaque | > 5 | sugarcane |
| **SRM-9** | Dull | Filamentous | Filamentous | Flat | Opaque | > 5 | sugarcane |
| **SRM-10** | Glistening | Irregular | Undulate | Umbonate | Translucent | > 5 | sugarcane |
| **SRM-11** | Dull | Round | Entire | Raised | Opaque | > 1 | sugarcane |
| **SRM-12** | Smooth | Round | Entire | Flat | Opaque | > 1 | sugarcane |
| **SRM-13** | Glistening | Round | Entire | Convex | Opaque | > 5 | sugarcane |
| **SRM-14** | Glistening | Irregular | Entire | Umbonate | Opaque | > 5 | sugarcane |
| **SRM-15** | Glistening | Round | Entire | Raised | Opaque | > 1 | sugarcane |
| **SRM-16** | Smooth | Round | Entire | Raised | Opaque | > 5 | sugarcane |
| **SRM-17** | Wrinkled | Irregular | Undulate | Umbonate | Opaque | > 5 | sugarcane |
| **SRM-18** | Smooth | Curled | Entire | Raised | Opaque | 5 | sugarcane |
| **SRM-19** | Glistening | Round | Entire | Convex | Opaque | > 1 | sugarcane |
| **SRM-20** | Glistening | Round | Entire | Raised | Opaque | 0.5 | sugarcane |
| **SRM-21** | Dull | Irregular | Undulate | Entire | Opaque | > 5 | sugarcane |
| **SRM-22** | Smooth | Curled | Entire | Undulate | Opaque | 0.5 | sugarcane |
| **SRM-23** | Glistening | Round | Entire | Entire | Opaque | > 5 | Rice |
| **SRM-24** | Glistening | Round | Undulate | Undulate | Opaque | > 1 | Rice |
| **SRM-25** | Smooth | Curled | Undulate | Entire | Translucent | > 1 | Rice |
| **SRM-26** | Dull | Round | Entire | Undulate | Opaque | > 5 | Rice |
| **SRM-27** | Wrinkled | Irregular | Undulate | Entire | Opaque | > 5 | Rice |
| **SRM-28** | Smooth | Curled | Entire | Raised | Opaque | > 1 | Rice |
| **SRM-29** | Glistening | Round | Entire | Convex | Opaque | > 1 | Rice |
| **SRM-30** | Dull | Round | Entire | Raised | Translucent | > 5 | Rice |
| **SRM-31** | Glistening | Round | Irregular | Entire | Opaque | > 1 | Rice |
| **SRM-32** | Dull | Round | Round | Raised | Opaque | > 1 | Rice |
| **SRM-33** | Wrinkled | Curled | Irregular | Undulate | Opaque | > 5 | Rice |
| SRM-34 | Glistening | Irregular | Curled | Entire | Opaque | > 5 | Rice |
| **SRM-35** | Smooth | Curled | Irregular | Raised | Translucent | > 1 | Rice |
| **SRM-36** | Wrinkled | Round | Round | Undulate | Opaque | > 1 | Rice |
| **SRM-37** | Smooth | Irregular | Irregular | Entire | Opaque | > 1 | Rice |
| **SRM-38** | Glistening | Round | Curled | Raised | Opaque | > 5 | Rice |
| **SRM-39** | Glistening | Round | Round | Entire | Opaque | > 1 | Rice |
| **SRM-40** | Wrinkled | Irregular | Round | Entire | Opaque | > 1 | Rice |
| **SRM-41** | Smooth | Irregular | Round | Raised | Opaque | > 1 | Rice |
| **SRM-42** | Wrinkled | Round | Irregular | Convex | Translucent | > 5 | Rice |
| **SRM-43** | Glistening | Irregular | Curled | Raised | Opaque | > 1 | Rice |
| **SRM-44** | Smooth | Irregular | Round | Raised | Opaque | > 1 | Rice |
