# Supplementary material for: Halotolerant rhizobacteria Pseudomonas pseudoalcaligenes and Bacillus subtilis mediate systemic tolerance in hydroponically grown soybean (Glycine max L.) against salinity stress
Source: PLoS One. 2020 Apr 16;15(4):e0231348. doi: 10.1371/journal.pone.0231348 (PMC7162512; doi:10.1371/journal.pone.0231348)
Supplement: S2 Table — (DOCX) [file pone.0231348.s004.docx]

**Table S2 Relative growth of bacterial strain on varying NaCl concentrations**

| **Sr. no.** | **1% NaCl** | **5% NaCl** | **10% NaCl** | **15% NaCl** | **20% NaCl** |
| --- | --- | --- | --- | --- | --- |
| **SRM-1** | + | + | + | + | - |
| **SRM-2** | + | + | - | - | - |
| **SRM-3** | + | + | + | + | + |
| **SRM-4** | + | + | + | + | - |
| **SRM-5** | + | + | + | - | - |
| **SRM-6** | + | + | - | - | - |
| **SRM-7** | + | + | - | - | - |
| **SRM-8** | + | + | - | - | - |
| **SRM-9** | + | + | + | + | + |
| **SRM-10** | + | + | - | - | - |
| **SRM-11** | + | + | + | - | - |
| **SRM-12** | + | + | + | - | - |
| **SRM-13** | + | + | - | - | - |
| **SRM-14** | + | + | + | + | - |
| **SRM-15** | + | + | - | - | - |
| **SRM-16** | + | + | + | + | + |
| **SRM-17** | + | + | - | - | - |
| **SRM-18** | + | + | - | - | - |
| **SRM-19** | + | + | - | - | - |
| **SRM-20** | + | + | + | + | + |
| **SRM-21** | + | + | + | - | - |
| **SRM-22** | + | + | - | - | - |
| **SRM-23** | + | + | + | - | - |
| **SRM-24** | + | + | - | - | - |
| **SRM-25** | + | + | + | + | - |
| **SRM-26** | + | + | - | - | - |
| **SRM-27** | + | + | - | - | - |
| **SRM-28** | + | + | - | - | - |
| **SRM-29** | + | + | + | - | - |
| **SRM-30** | + | + | - | + | - |
| **SRM-31** | + | + | - | - | - |
| **SRM-32** | + | + | + | - | - |
| **SRM-33** | + | + | - | - | - |
| SRM-34 | + | + | + | - | - |
| **SRM-35** | + | + | - | - | - |
| **SRM-36** | + | + | + | - | - |
| **SRM-37** | + | + | - | - | - |
| **SRM-38** | + | + | - | - | - |
| **SRM-39** | + | + | - | + | - |
| **SRM-40** | + | + | + | - | - |
| **SRM-41** | + | + | - | - | - |
| **SRM-42** | + | + | - | - | - |
| **SRM-43** | + | + | - | + | - |
| **SRM-44** | + | + | + | - | - |
